# Shark Antibody Variable Domains Rigidify Upon Affinity Maturation—Understanding the Potential of Shark Immunoglobulins as Therapeutics

**DOI:** 10.3389/fmolb.2021.639166

**Published:** 2021-04-20

**Authors:** Monica L. Fernández-Quintero, Clarissa A. Seidler, Patrick K. Quoika, Klaus R. Liedl

**Affiliations:** Department of General, Inorganic and Theoretical Chemistry, Center for Molecular Biosciences Innsbruck (CMBI), University of Innsbruck, Innsbruck, Austria

**Keywords:** shark antibodies, V_*NAR*_, affinity maturation, binding mechanisms, conformational selection, encounter complex, binding interfaces

## Abstract

Sharks and other cartilaginous fish are the phylogenetically oldest living organisms that have antibodies as part of their adaptive immune system. As part of their humoral adaptive immune response, they produce an immunoglobulin, the so-called immunoglobulin new antigen receptor (IgNAR), a heavy-chain only antibody. The variable domain of an IgNAR, also known as V_*NAR*_, binds the antigen as an independent soluble domain. In this study, we structurally and dynamically characterized the affinity maturation mechanism of the germline and somatically matured (PBLA8) V_*NAR*_ to better understand their function and their applicability as therapeutics. We observed a substantial rigidification upon affinity maturation, which is accompanied by a higher number of contacts, thereby contributing to the decrease in flexibility. Considering the static x-ray structures, the observed rigidification is not obvious, as especially the mutated residues undergo conformational changes during the simulation, resulting in an even stronger network of stabilizing interactions. Additionally, the simulations of the V_*NAR*_ in complex with the hen egg-white lysozyme show that the V_*NAR*_ antibodies evidently follow the concept of conformational selection, as the binding-competent state already preexisted even without the presence of the antigen. To have a more detailed description of antibody–antigen recognition, we also present here the binding/unbinding mechanism between the hen egg-white lysozyme and both the germline and matured V_*NAR*_s. Upon maturation, we observed a substantial increase in the resulting dissociation-free energy barrier. Furthermore, we were able to kinetically and thermodynamically describe the binding process and did not only identify a two-step binding mechanism, but we also found a strong population shift upon affinity maturation toward the native binding pose.

## Introduction

Cartilaginous fish, such as sharks, rays, chimeras, and skates, are the phylogenetically oldest group of animals having a canonical adaptive immune system (Cooper and Alder, 2006; Dooley and Flajnik, 2006; Flajnik and Kasahara, 2010). Thus, shark antibodies can provide insights into the molecular evolution of the immune system (Feige et al., 2014). For 500 million years, sharks have dominated the oceans as predators. During that time, their immune system, the oldest adaptive immunity known, evolved and already produced key parts of the immune system, such as T cells, B cells, and major histocompatibility complexes (MHCs), which can also be found in mammals (Frommel et al., 1971; Criscitiello et al., 2006; Feige et al., 2014; Flajnik, 2018). However, sharks have developed unique structural and immunological features, which cannot be found in humans or other mammals, except in camelids. Additionally, it has been shown that immunoglobulin new antigen receptors (IgNARs) reveal the highest potential for antigen-driven affinity maturation, compared with other Ig isotypes in sharks (Diaz et al., 2002; Feige et al., 2014).

**FIGURE 1:**
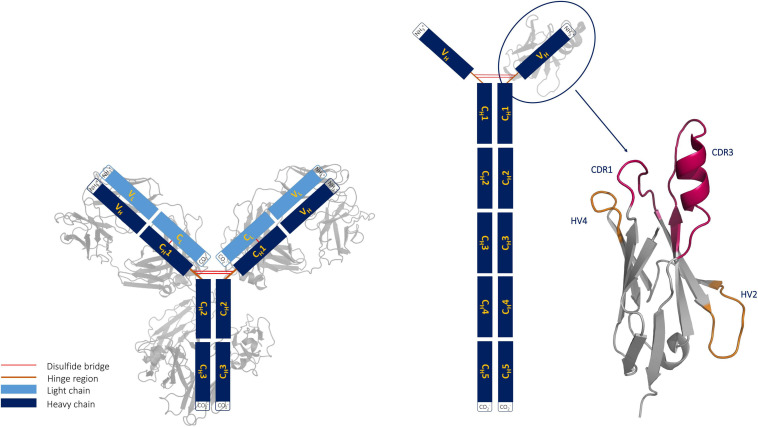
Structural comparison of an immunoglobulin (IgG) structure with an IgG new antigen receptor (IgNAR). The structure of a V_*NAR*_ with its unique binding site geometry is depicted next to the IgNAR.

Shark immunoglobulins are comprised of heavy-light chain isotypes, known as IgM and IgW, and one heavy chain homodimeric isotype called IgNAR (Hsu, 2016). The IgNAR antibodies are disulfide-bonded homodimers. The two heavy chains dimerize *via* five constant domains, while the two variable domains (V_*NAR*_s) are unpaired, forming the tips of the IgNARs (Roux et al., 1998; Diaz et al., 2002; Zielonka et al., 2015). Furthermore, it has been shown that dimerization is not required for high-affinity antigen binding of V_*NAR*_s, suggesting that ancient V_*NAR*_s were already functional single-domain antigen-binding domains compared with the homodimeric IgNARs found in modern sharks. Even though shark V_*NAR*_ and camelid V_*H*_H antibodies have similar structural features, they differ in their evolution, as camelid V_*H*_H evolved from an IgG by simultaneously losing the light chain and C_*H*_1 domain of the heavy chain (Figure 1; Clem and Leslie, 1982; Barelle et al., 2009; English et al., 2020).

Shark antibodies evolved under challenging conditions, which makes them particularly stable. Apart from their high stability and solubility, V_*NAR*_s have the ability to recognize and bind hidden functional sites of a target antigen, making them especially attractive as novel therapeutics for human diseases (Barelle et al., 2009; English et al., 2020). A lysozyme-binding antibody variable fragment (Fv) (PDB accession code: 2EIZ) was recently compared with a nurse shark structure complexed with lysozyme (PDB accession code: 1T6V) (Stanfield et al., 2004; Nakanishi et al., 2008). The study revealed that in contrast to the antibody Fv, the V_*NAR*_ can recognize the buried substrate pocket of lysozyme with its extended CDR3 loop. V_*NAR*_ fragments contain only two complementarity-determining region (CDR) loops and are still able to target antigens through a single variable domain. To compensate for this reduced size (∼13 kDa), the binding site is characterized by a long and structurally complex CDR3 loop. Consequently, the highest diversity in length, sequence, and structure in V_*NAR*_s is located in the CDR3 loop; however, the number and position of cysteine residues also contribute to determining the structural diversity of V_*NAR*_s (Streltsov et al., 2005). In general, V_*NAR*_ domains consist of two β sheets, which are stabilized by a disulfide bond between two canonical cysteine residues (21C and 82C) located in the framework. Based on the number and position of additional cysteine residues, four types of naturally occurring IgNAR variable domains have been reported (Roux et al., 1998; Rumfelt et al., 2001; Streltsov et al., 2005; Matz and Dooley, 2019). The CDR3 loops of both type I and type II VNARs have extended CDR3 loops, in a so-called “upright” position, which allows to reach and bind buried epitopes. V_*NAR*_ domains comprise longer CDR3 loops (up to 40 amino acids), compared with CDR3 loops in humans, and lack the CDR2 loop, which generally plays an important role in IgG and camelid V_*H*_H antibodies. Instead, V_*NAR*_s contain other CDR2 like regions, which are the hypervariable loops 2 and 4 (HV2 and HV4, respectively). The importance of the HV4 loop for antigen recognition has been reported for T-cell receptor variable β domains (Fernández-Quintero et al., 2020g).

In this study, we investigate the consequences and effects of somatic hypermutations of a nurse shark PBLA8 antibody upon affinity maturation and characterize the respective antibody–antigen-binding processes. The PBLA8 is a type II VNAR clone, which is part of a phage-display library derived from a lysozyme-immunized nurse shark, also known as *Ginglymostoma cirratum*. Type II VNAR antibodies are characterized by their specific, stabilizing disulfide bonds in the CDR3 and CDR1 loops. Both the ancestral and the matured PBLA8 clones were derived from the same ancestral B cell. The matured PBLA8 antibody contains 13 somatic mutations, four in the CDR1, two in the HV2 loop, and one each in the HV4 and the CDR3 loops.

## Results

**FIGURE 2:**
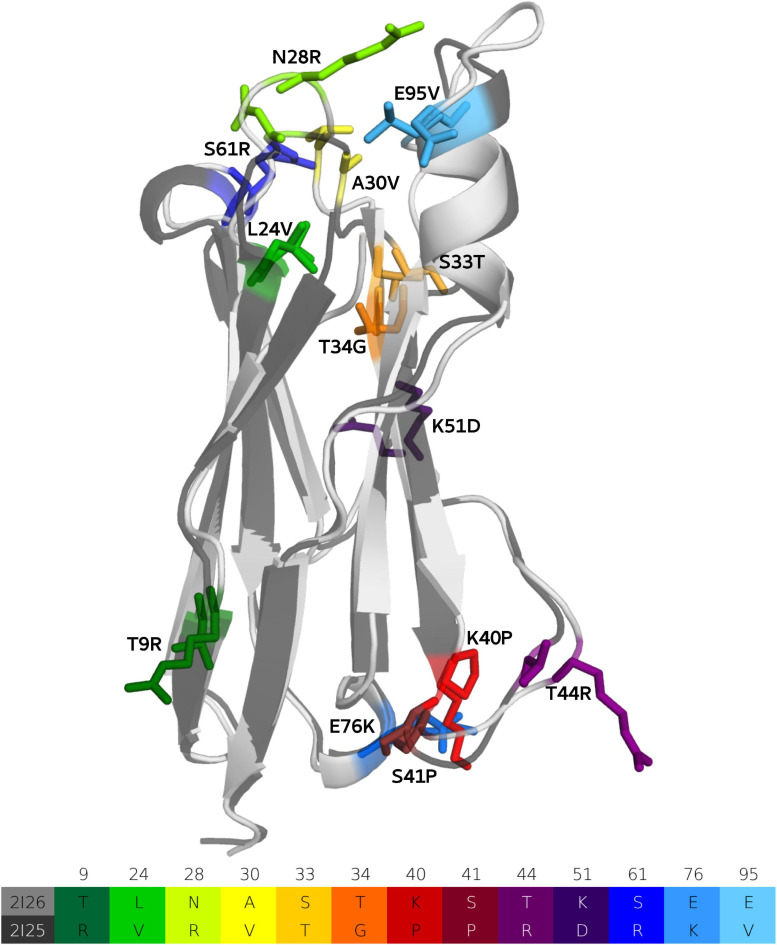
Sequence alignment of the 13-point mutations and structural comparison of the ancestral and matured V_*NAR*_ domains are illustrated and color coded, respectively.

We use a well-established protocol combining enhanced sampling techniques with classical molecular dynamics simulations to elucidate the affinity maturation process (Fernández-Quintero et al., 2019b, 2020c,g) and describe the antigen-binding mechanism of V_*NAR*_ antibodies with the antigen, lysozyme. Four crystal structures of the investigated V_*NAR*_, before and after affinity maturation, and with and without the presence of the antigen (PDB accession codes: 2I26, 2I27, 2I25, and 2I24, respectively) (Stanfield et al., 2007) were available and were used as starting structures for metadynamics simulations. The matured PBLA8 antibody clone contains in total 13 mutations, compared with its germline ancestor. Figure 2 illustrates both the naive (light gray) and the affinity matured (dark gray) V_*NAR*_ domains, including a sequence-comparison of the mutated residues, color-coded in the table below and in the structure. As described in the methods section, we performed 1 μs of metadynamics simulations for all four available crystal structures to enhance the sampling of the CDR1 and CDR3 loops of both the naive and the matured V_*NAR*_s. We did not delete the antigen in our simulations, to be able to structurally characterize the antigen-binding process. Thus, to identify the influence of the 13 somatic hypermutations on both the conformational space and on the antigen-binding process, the dynamic nature, and the conformational diversity of the V_*NAR*_s has to be considered.

To quantify the resulting flexibility between the naive and the matured V_*NAR*_s, we performed hierarchical clustering of all four metadynamics simulations individually on the CDR1 and CDR3 loops of both the naive and the matured V_*NAR*_s with and without the antigen present, using the same Root Mean Square Deviation (RMSD) cut-off criterion of 1.5 Å.

**FIGURE 3:**
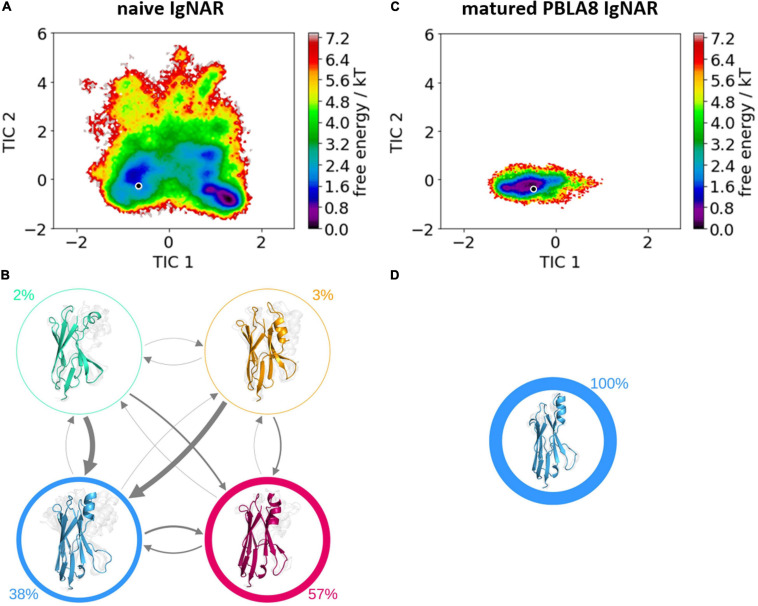
Free energy surfaces and Markov-state models of the apo naive and matured V_*NAR*_ CDR3 and CDR1 loops. **(A)** Free energy surface of the naive V_*NAR*_ CDR3 and CDR1 loops, the starting X-ray structure (PDB accession code: 2I27) is depicted as a black dot. **(B)** Results of the Markov-state models with the respective macrostate probabilities. The thickness of the arrows denotes the transition timescale and the width of the surrounding circle represents the state population. **(C)** Free energy surface of the matured V_*NAR*_ CDR3 and CDR1 loops projected into the same coordinate system as the naive V_*NAR*_ and the starting crystal structure is also illustrated as a black dot (PDB accession code: 2I24). **(D)** Agreement with the obtained free energy surface only one macrostate.

**FIGURE 4:**
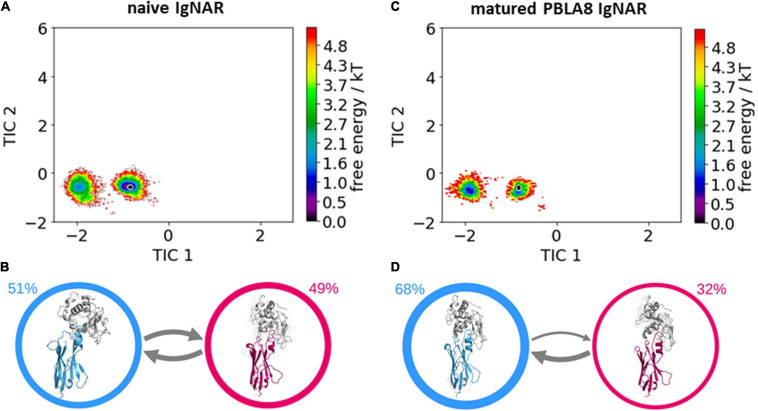
Free energy surfaces and Markov-state models of the complexed naive and matured V_*NAR*_ CDR3 and CDR1 loops. **(A)** Free energy surface of the naive V_*NAR*_, the starting x-ray structure (PDB accession code: 2I26) is depicted as a black dot. **(B)** Results of the Markov-state models with the respective macrostate probabilities. The thickness of the arrows denotes the transition timescale and the width of the surrounding circle represents the state population. **(C)** Free energy surface of the matured V_*NAR*_ CDR3 and CDR1 loops projected into the same coordinate system as the naive V_*NAR*_ and the starting crystal structure is also illustrated as a black dot (PDB accession code: 2I25). **(D)** Agreement with the obtained free energy landscape two macrostates and again the transition timescales and state populations are represented by the thickness of the arrows and the width of the circles, respectively.

Table 1 summarizes the resulting numbers of clusters and also includes additional clustering results using different input criteria. Independent of the input features and the cut-off criterion applied for the clustering, we observe a substantial decrease in the number of clusters as a consequence of affinity maturation. We also observe that antigen-binding results in a decrease of flexibility in the binding site, reflected in a smaller number of clusters for the different input criteria, except for the HV4 loop. The reason for this increase in flexibility of the HV4 loop upon binding is that this loop is not directly involved in the antigen-binding process. Therefore, the rigidification of the CDR1 and CDR3 loops allows a higher variability of the HV4 loop. To reconstruct the kinetics and thermodynamics of the different CDR loop rearrangements, we used the obtained cluster representatives as starting structures for every 100 ns of classical molecular dynamics simulations. These trajectories were then used to construct a time-lagged independent component analysis (tICA) and a Markov-state model based on the backbone torsions of the CDR1 and CDR3 loops. Figure 3 illustrates the Markov-state models and the reweighted free energy surfaces of the naive and the matured PBLA8 V_*NAR*_s, which were performed without the antigen. From the resulting free energy surfaces projected into the combined coordinate system in Figures 3A,C, we observed a substantial rigidification of conformational space upon affinity maturation, which is accompanied by a strong population shift toward the binding competent state. Interestingly, even without the presence of the antigen, we find for both variants that the binding competent state already preexists in the captured CDR loop ensembles in solution with varying probabilities. The Markov-state models depicted in Figures 3B,D, clearly confirm this population shift upon affinity maturation. While the matured PBLA8 antibody shows only one deep and narrow minimum, the naive antibody results in four different CDR loop macrostates with conformational transitions of the CDR loops in the microsecond timescale. Figure 4 illustrates the free energy landscapes and the respective Markov-state models of the naive and matured V_*NAR*_ domains, simulated with the antigen present. Furthermore, these free energy surfaces are also projected into the same coordinate system as shown in Figure 3. These results confirm the strong population shift upon affinity maturation toward the binding competent state. Additionally, the naive V_*NAR*_ strongly supports the conformational selection paradigm, as the binding competent state already preexists with lower probability without the presence of the antigen and was selected as the dominant solution structure upon binding.

**TABLE 1:** Summary of all clustering results and overview of the aggregated simulation times.

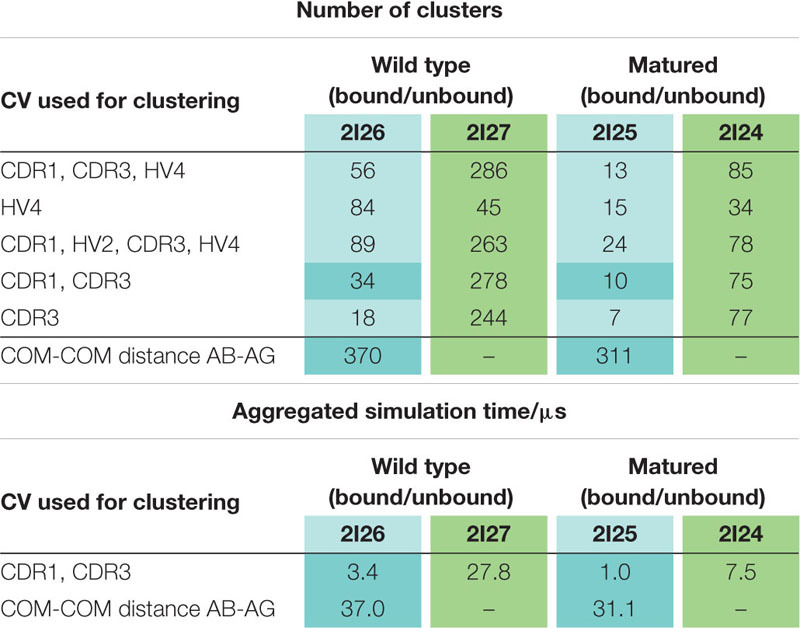

*The top table shows the number of clusters resulting from different clustering criteria. The number of clusters, which were used as starting structures for every 100 ns of molecular dynamics simulations, is highlighted in cyan. The bottom table illustrates the aggregated simulation time, respectively.*

**FIGURE 5:**
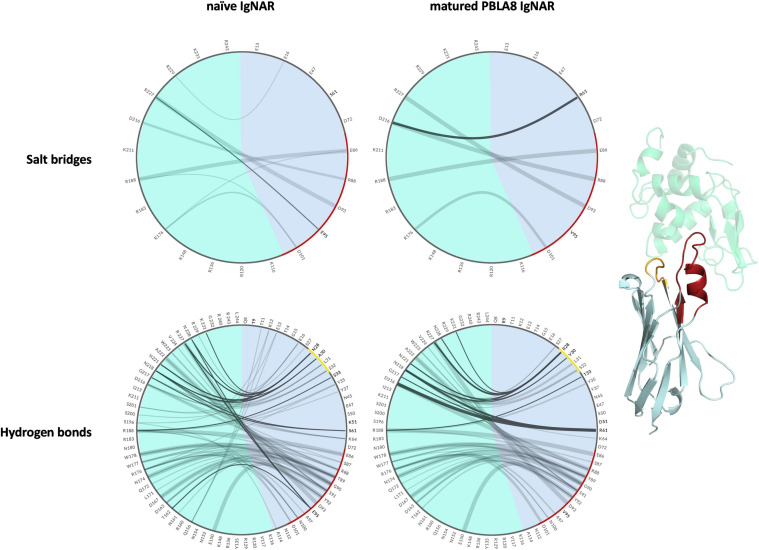
Visualization of the salt bridges and hydrogen bond interactions between lysozyme and the naive and matured V_*NAR*_ domains represented as flare plots. The residues of the antibody are colored in blue, while the antigen is depicted in green. The color-coding corresponds to the structure illustrated next to the flare plots. The CDR3 loop is colored red and the CDR1 loop is depicted in yellow. The mutated residues are shown in bold.

To compare the interactions of the naive and the matured V_*NAR*_ with the antigen and to structurally elucidate the antigen-binding process, we visualized the different types of contacts (hydrogen bonds and salt bridges) as individual flare plots (Figure 5). The thickness of the lines in these plots represents the duration of the contacts. The flare plot is divided into two colors, blue for the antibody and green for the antigen. The CDR1 and CDR3 loops are also highlighted in yellow and red, respectively. The numbering and position of the residues can directly be compared between the naive and matured V_*NAR*_. These facilitate the comparison between the two variants. In agreement with the observed rigidification upon affinity maturation in the presence of the antigen, the decrease in flexibility of the matured V_*NAR*_ can be structurally explained by the substantially higher number of contacts and long-lasting interactions formed between the hen egg-white lysozyme and the matured PBLA8 V_*NAR*_.

To further structurally and mechanistically characterize the antigen-binding process, we again performed metadynamics simulations, but used the distance between the center of masses of the antigen and the antibody as a collective variable, ensuring the minimal distortion of the binding interface. These simulations allow us to cover a broad range of unbinding pathways and to elucidate the antigen-binding process in detail. As mentioned in the methods section, three individual runs of metadynamics simulations were started with different initial velocities. We combined and clustered the simulations on the center of mass distances between the antibody and the antigen for each variant separately. The resulting cluster representatives were used as starting structures for short classical molecular dynamics simulations to allow an unbiased view of the mechanism involved in antibody–antigen recognition and binding.

**FIGURE 6:**
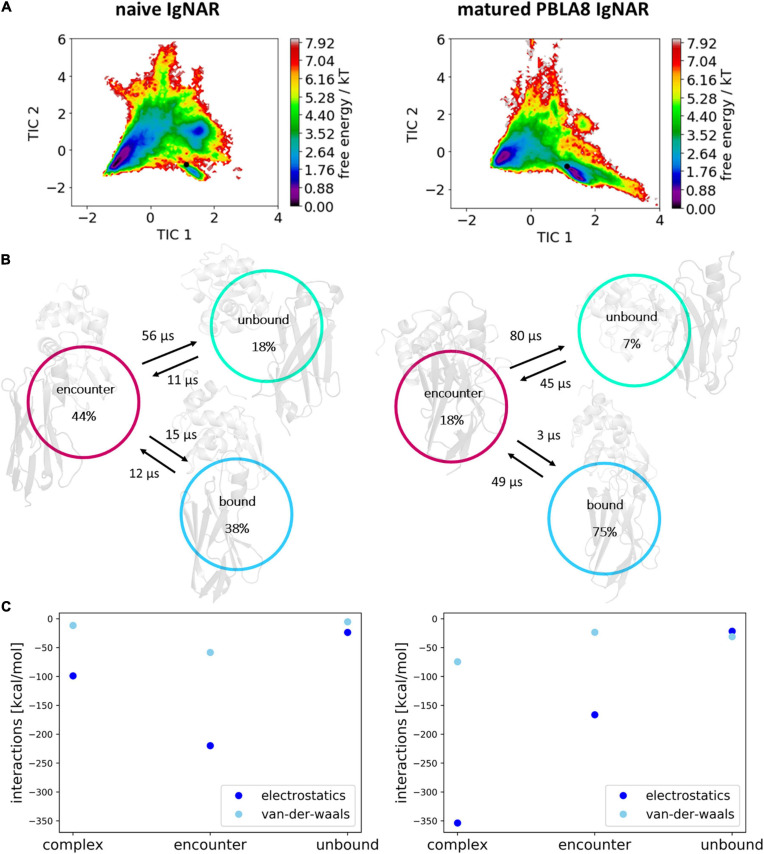
Free energy surfaces, Markov-state models, and interaction energies within the obtained three metastable states (complex, encounter and unbound). **(A)** Free energy surfaces of the observed unbinding pathways of the naive and matured V_*NAR*_ domains. The Markov-state model is depicted in panel **(B)** showing the state probabilities and transition timescales between the three identified states. **(C)** Average electrostatic and Van der Waals interactions at the binding interface of the antibody and the antigen for the complex, encounter, and unbound state.

To identify kinetically stable states along the binding pathway, we apply tICA on the inverse distances of the native contacts. We chose inverse distances as they are well suited to distinguish small differences between conformations where the V_*NAR*_ and the antigen are close, but not overemphasizing the differences in unbound conformations (big distances, small inverse distances). Besides, inverse distances are functionally closer to potential energies. In Figure 6A, the resulting free energy surfaces and the Markov-state models of the antigen–antibody binding pathways of both the naive and the matured V_*NAR*_s are illustrated. For these two antibody–antigen complexes, we observe three metastable states along the binding pathway. The main difference between the naive and the matured antibody is the populations of these three metastable states. Particularly interesting is the strong population shift upon affinity maturation toward the binding competent conformation, compared with the naive V_*NAR*_. Before unbinding in both variants, a so-called “encounter complex” could be identified, which already shows a significantly higher number of electrostatic interactions, compared with the completely unbound conformations (Figure 6C). The encounter complex of the naive V_*NAR*_ is even more dominated by electrostatics compared with the complexed state, which is in agreement with the obtained free energy surface and the Markov-state model showing that the encounter complex is the highest populated state. The encounter complex formation is dominated by ionic interactions of Glu 86 with lysozyme Arg 73 (corresponding to R188) (occurrence 60%) and Arg 88 with lysozyme Asp 101 (corresponding to D216) (occurrence 15%) and hydrogen bond interactions of Tyr 89 with lysozyme Trp 63 (corresponding to W178) (occurrence 30%) and lysozyme Asp 52 (corresponding to D167) (occurrence 6%). Additionally, Tyr 92 forms a hydrogen bond with lysozyme Asp 48 (corresponding to D163) (occurrence 10%).

Figure 6C clearly shows that the unbinding process of the matured V_*NAR*_ is strongly governed by electrostatics, while Van der Waals interactions play only a minor role in the association of the antibody and the antigen in the transition from the unbound state to the formation of the encounter complex. Especially interesting is that the formation of the encounter complex of the matured V_*NAR*_ is favored by ionic interactions formed by the mutated residue Asp 51, which was a Lys 51 before maturation. Asp 51 forms ionic interactions with lysozyme Arg 21 (corresponding to R136) (occurrence 15%) and lysozyme Lys 96 (corresponding to K211) (occurrence 14%) and makes an additional hydrogen bond with lysozyme Tyr 20 (corresponding to Y135) (occurrence 20%). Furthermore, we observe hydrogen bond interactions of Ser 48 with lysozyme Asp 101 (occurrence 30%). However, Figure 6C also illustrates that the Van der Waals interactions have a more prominent role in the transition from the encounter complex to the native complexed state.

## Discussion

The rise of antibodies as therapeutics has motivated numerous studies to characterize and understand the antibody binding interface as a pre-requisite for rational antibody design and engineering (MacCallum et al., 1996; Schmidt et al., 2013; Di Palma and Tramontano, 2017; Fernández-Quintero et al., 2019a,b, 2020d,f). Compared with conventional antibodies, small antibodies such as nanobodies and V_*NA*__*R*_s are more stable and more soluble. Additionally, they can work inside cells as their small size allows them to wend into tissues and they can recognize cryptic epitopes (Griffiths et al., 2013).

The transfer across the blood–brain barrier (BBB) remains a challenge in the development of biotherapeutics that affect the central nervous system. However, it has already been reported that V_*NAR*_s can reach the brain, making them especially attractive for use as therapeutic, diagnostic, or transport molecules. Additionally, just recently, a V_*NAR*_ targeting the transferrin receptor 1 (TfR1) is transported through the BBB into the brain parenchyma, highlighting the importance of V_*NAR*_s as they can shuttle molecules across the BBB (Stocki et al., 2019).

Thus, structurally characterizing the peculiar antibody-binding site of V_*NAR*_s and understanding antibody–antigen recognition is crucial for the design and engineering of these outstanding proteins. In this study, we thermodynamically and kinetically characterize CDR loop ensembles in solution before and after affinity maturation and explain the observed rigidification in atomic detail. However, apart from the decrease in conformational space, the underlying binding mechanisms were also investigated, including a description of the fundamental factors that contribute to antigen recognition and binding.

Conformational rearrangements in the paratope, as well as binding and unbinding events of an antigen, can occur in the microsecond to second timescale, which exceeds routinely performed simulation times by far. To enhance the efficiency of the sampling, we used metadynamics simulations to cover conformational transitions between different CDR loop conformations, but also to capture conformations along the path between the complex and dissociated V_*NAR*_–lysozyme complex.

The comparison of the obtained free energy landscapes of the naive with the matured V_*NAR*_s (Figure 3) without the presence of the antigen clearly shows a substantial rigidification upon affinity maturation as a consequence of 13-point mutations. This broader conformational space of the CDR loops is governed by the higher flexibility of the CDR3 loop in the naive V_*NAR*_, compared with the matured PBLA8 V_*NAR.*_ The stabilization of the CDR3 loop originates from a salt bridge and a hydrogen bond formed between an Arg28 in the CDR1 and an Asp93 in the CDR3 loop. Both the salt bridge and the hydrogen bond interactions are present in nearly all frames of the simulations (95%). The higher flexibility of the naive V_*NAR*_ can be explained by the absence of these interactions, as residue 28 is an asparagine before maturation, which only forms a hydrogen bond with Asp93 in 2% of the frames. Upon antigen binding, the stabilizing intramolecular network of interactions within the matured PBLA8 V_*NAR*_ of Asp93 with Arg28 and Asp93 with Ser43 (12% of occurrence) is changed to a salt bridge of Asp93 with lysozyme Arg112 (corresponds to R227 in Figure 5). Another salt bridge and hydrogen bond interaction between the matured PBLA8 V_*NAR*_ and lysozyme could be identified—Arg61 and lysozyme Asp 101 (corresponding to D216) and Asn 103 (corresponding to N218) strongly interact with each other, which is unique for the matured variant. Before binding, the Arg 61 was interacting with the Asn 60 (15% of occurrence) and with the backbone of Thr 58 (45% occurrence). Overall, the duration and number of contacts between the antibody and lysozyme are much higher in the matured PBLA8 V_*NAR*_, compared with the naive V_*NAR*_ (Supplementary Figures 1–3). Additionally, the residues Arg 61, Asp 51, and D101 in the matured V_*NAR*_ turn out to be key determinants for molecular recognition of the antigen. Astonishingly, Asp 93 can form equally strong interactions with Asp 101 in both the matured and the naive V_*NAR*_. However, Asp 93 contributes together with Arg 28 substantially to an intramolecular interaction network, contributing to the significant increase in flexibility before binding upon affinity maturation.

Furthermore, the structural changes of the CDR3 and CDR1 loops upon antigen binding have been reported to follow the induced fit theory, as it was assumed that the observed conformational changes in the CDR loops were induced by antigen binding (Koshland Daniel, 1995; Stanfield et al., 2007). However, we find that within the obtained dynamic apo ensemble of the naive V_*NAR*_ the binding competent conformation already preexists without the presence of the antigen (Ma et al., 1999; Tsai et al., 1999; Fernández-Quintero et al., 2020e). As the antigen recognizes and binds to this conformation, we observe a strong population shift toward the binding competent conformation (Figure 3). The free energy surface of the affinity-matured PBLA8 V_*NAR*_ simulated without antigen exhibits only one deep narrow minimum, in which the binding competent state already preexists. As the matured PBLA8 V_*NAR*_ rigidifies substantially upon affinity maturation, with only small structural rearrangements upon antigen recognition being observed, the binding process can be described as lock-and-key binding. This has already been reported in 1997, where significant conformational changes occurred in the germline antibody upon binding, while the matured antibody was identified to bind the antigen by a lock-and-key-fit mechanism (Koshland Daniel, 1995; Wedemayer et al., 1997).

To better understand the antibody–antigen recognition and the effect of affinity maturation on the complex formation, we investigated the detailed binding and unbinding pathways of the naive and the matured PBLA8 V_*NAR*_ with the antigen lysozyme (Figure 6). Our results clearly show that the pathway of the binding process can be described as a two-step mechanism. The most critical step represents the association of the binding partners and the formation of the encounter complex which is characterized by a protein–protein interface dominated by electrostatic interactions (Schreiber and Fersht, 1996; Vijayakumar et al., 1998; Sheinerman et al., 2000; Frisch et al., 2001). At this stage, the protein–protein interface is still partially solvated and contains non-optimal sidechain orientations and interactions (Horn et al., 2009; Kahler et al., 2020). Electrostatic interactions are the driving force in directing the binding and pulling of the antibody–antigen interface together. Thereby, electrostatics may also contribute to the early discrimination between potential binding partners. While the encounter complex features the pre-aligned binding partners, further side-chain rearrangements and closer approaching of the two binding partners results in desolvation (Camacho et al., 2000) and a more prominent role of the Van der Waals interactions (Figure 6).

**FIGURE 7:**
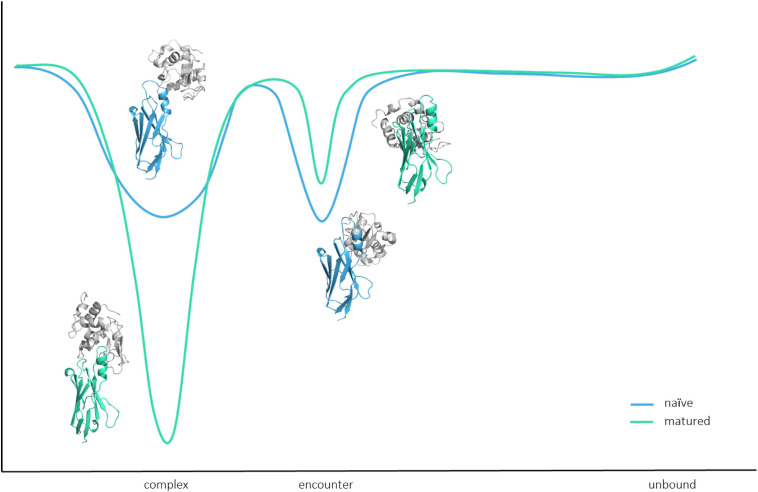
Schematic summary and representation of the binding pathway. Upon affinity maturation, the native complex state becomes the most probable state, while the encounter complex is favored in the binding pathway of the naive antibody.

Figure 6 shows that for both the naive and the matured V_*NAR*_, an energy barrier between the encounter complex and the complex state, which prohibits a fast transition to the native complex state (Alsallaq and Zhou, 2007). The encounter complex in the naive V_*NAR*_–lysozyme binding pathway is the highest populated state and also shows the highest electrostatic interaction energy. This observation is confirmed by the high number of electrostatic interactions formed in the encounter complex. Moreover, we find that especially in the naive VNAR tyrosine residues contribute substantially to the formation and stabilization of the encounter complex. Tyrosine residues have been shown to play a privileged role in antigen recognition by contributing substantially to mediating molecular contacts in the binding interfaces (Koide and Sidhu, 2009). The hydroxyl sidechain makes tyrosine significantly more hydrophilic compared with other hydrophobic amino acids. At the same time, increased hydrophilicity may result in less specific binding in the unbound state, which might be a key characteristic of a naive repertoire, which can still be exposed to a diverse number of antigenic surfaces (Koide and Sidhu, 2009). Excitingly, we observe a strong population shift upon affinity maturation toward the binding competent state (naive 38%–matured 75% population). For the matured V_*NAR*_, the formation of the encounter complex, as well as the optimization and transition to the native complex state, are dominated by electrostatics (Tworowski et al., 2005). Figure 7 schematically represents and summarizes the observed binding mechanism for both the naive and the matured antibody. While in the naive antibody, the formation of the encounter complex is energetically more favored compared with the native complex, the matured V_*NAR*_ reveals a strong population shift toward the complex state. Thus, affinity maturation does not only result in a decrease in conformational diversity of the CDR loops but strongly favors the formation of the native complex, which is governed by both electrostatic and Van der Waals interactions.

## Conclusion

In this study, we structurally and functionally characterized the antigen-binding site of V_*NAR*_s upon affinity maturation. We observed that not only the CDR1 and CDR3 loops, which are directly involved in the antigen-binding process, but also the whole V_*NAR*_s rigidify upon maturation, as a consequence of the 13-point mutations. The obtained free energy surface of the naive V_*NAR*_ is broad and shallow, while the matured PBLA8 V_*NAR*_ only shows one deep and narrow minimum. This resulting rigidification is accompanied by a strong population shift upon affinity maturation. Additionally, we evidently see that the naive V_*NAR*_ variant follows the concept of conformational selection, while antigen recognition of the matured V_*NAR*_ can be described as a lock-and-key binding.

Furthermore, we provide a two-step binding mechanism and describe in detail the driving forces of antibody–antigen association. Thereby, we present a comprehensive model of antibody–antigen recognition. Apart from identifying key determinants for antigen recognition, we also elucidate the affinity maturation mechanism, as we observe a significant population shift from the naive to the matured variant toward the binding competent complex state, which is represented by a deep and narrow minimum in the free energy surface. Thus, these results have broad implications for the rational design of new antigen receptors, i.e., V_*NAR*_, since they provide a detailed characterization of the intra-and-intermolecular changes upon affinity maturation. Additionally, these insights presented on the binding pathways in different stages of affinity maturation, combine a variety of fundamental concepts in molecular recognition which can be used to improve protein–protein docking, and consequently, the engineering of specific and stable antibody–antigen complexes.

## Materials and Methods

A previously published method characterizing the CDR loop ensembles upon antigen binding in solution (Fernández-Quintero et al., 2019a,b, 2020a,b,e,g) was used to investigate the conformational diversity of CDR3 and CDR1 loops of V_*NAR*_ variants in different stages of affinity maturation. Experimental structure information was available for the naive and the matured V_*NAR*_s, crystallized with and without the antigen, hen egg-white lysozyme. The PDB accession codes for the naive V_*NAR*_s with and without the presence of the antigen are 2I26 and 2I27, respectively (Stanfield et al., 2007). The crystal structures for the matured variant with and without the antigen can be found in the PDB with the accession codes 2I25 and 2I24. All four available x-ray structures were used as starting structures for molecular dynamics simulations. The starting structures for simulations were prepared in Molecular Operating Environment (Chemical Computing Group, version 2020.01) using the Protonate3D tool (Labute, 2009; Chemical Computing Group, 2020). To neutralize the charges, we used the uniform background charge (Hub et al., 2014; Case et al., 2020). Using the tleap tool of the AmberTools20 (Roe and Cheatham, 2013; Case et al., 2020) package, the crystal structures were soaked in cubic water boxes of TIP3P water molecules with a minimum wall distance of 10 Å to the protein (Jorgensen et al., 1983; El Hage et al., 2018; Gapsys and de Groot, 2019). For all simulations, parameters of the AMBER force field 14SB were used (Maier et al., 2015). The V_*NAR*_ variants were carefully equilibrated using a multistep equilibration protocol (Wallnoefer et al., 2011).

### Metadynamics Simulations

To enhance the sampling of the conformational space, well-tempered metadynamics simulations (Barducci et al., 2008, 2010; Ilott et al., 2013; Biswas et al., 2018) were performed in GROMACS (Pronk et al., 2013; Abraham et al., 2015) with the PLUMED 2 implementation (Tribello et al., 2014). As collective variables, we used a linear combination of sine and cosine of the ψ torsion angles of the CDR1 and CDR 3 loops calculated with functions MATHEVAL and COMBINE implemented in PLUMED 2 (Tribello et al., 2014). As discussed previously, the ψ torsion angle captures conformational transitions comprehensively (Ramachandran et al., 1963). The decision to include the ψ torsion angles of these two loops is based on their strong involvement in the binding to the antigen as evident from the x-ray structure of the complex. The simulations were performed at 300 K in an NpT ensemble. The height of the Gaussian was determined according to the minimal distortion of the V_*NAR*_ systems, resulting in a Gaussian height of 10 kJ/mol, and a width of 0.3 rad. Gaussian deposition occurred every 1,000 steps and a bias factor of 10 was used. Metadynamics simulations measuring 1 μs were performed for each available V_*NAR*_ crystal structure. The resulting trajectories were clustered in cpptraj (Roe and Cheatham, 2013; Case et al., 2020) using the average linkage hierarchical clustering algorithm with a distance cut-off criterion of 1.5 Å resulting in a large number of clusters (Table 1). The cluster representatives for the matured and the naive variants, both with and without the antigen present, were equilibrated and simulated for 100 ns using the AMBER 20 simulation package.

To further elucidate the detailed binding mechanism and to investigate the effects of point mutations on the antigen-binding process, we performed additional non-well-tempered metadynamics simulations using the distance between the two centers of masses of the V_*NAR*_ and the antigen as collective variables (Alessandro and Gervasio, 2008; Barducci et al., 2011). We used a Gaussian height of 1 kJ/mol and width of the Gaussian of 0.1 nm. An additional Gaussian function has also been introduced every 1,000 simulation steps. Three individual runs of both the matured and the naive antibody were performed for 10 ns of simulation time, each. The obtained trajectories were clustered using the distance between the two centers of masses as a clustering criterion with a distance of 1.5 Å. To reconstruct the thermodynamics and kinetics of the binding process, the resulting large number of cluster representatives were again equilibrated and simulated for 100 ns each using the AMBER 20 simulation package (Table 1).

### Molecular Dynamics Simulations

Molecular dynamics simulations were performed in an NpT ensemble using pmemd.cuda (Salomon-Ferrer et al., 2013). Bonds involving hydrogen atoms were restrained by applying the SHAKE algorithm (Miyamoto and Kollman, 1992), allowing a time step of 2 fs. The atmospheric pressure of the system was preserved by weak coupling to an external bath using the Berendsen algorithm (Berendsen et al., 1984). The Langevin thermostat (Doll et al., 1975; Adelman and Doll, 1976) was used to maintain the temperature during simulations at 300 K.

Additionally, a tICA was performed using the python library PyEMMA 2 employing a lag time of 10 ns (Scherer et al., 2015; Pérez-Hernández and Noé, 2016). Thermodynamics and kinetics were calculated with a Markov-state model (Bowman et al., 2014; Chodera and Noé, 2014) using PyEMMA 2, which uses the k-means clustering algorithm (Likas et al., 2003) to define microstates and the PCCA + clustering algorithm (Röblitz and Weber, 2013) to coarse-grain the microstates to macrostates. PCCA + is a spectral clustering method, which discretizes the sampled conformational space based on the eigenvectors of the transition matrix. The sampling efficiency and the reliability of the Markov-state model (e.g., defining optimal feature mappings) can be evaluated with the Chapman–Kolmogorov test (Karush, 1961; Miroshin, 2016), using the variational approach for Markov processes (Wu and Noé, 2017) and by taking into account the fraction of states used, as the network states must be fully connected to calculate probabilities of transitions and the relative equilibrium probabilities. To capture and quantify the CDR loop rearrangements of the V_*NAR*_ variants, we constructed Markov-state models based on the backbone torsions of the CDR1 and CDR3, defined 150 microstates using the k-means clustering algorithm, and applied a lag time of 10 ns.

To reconstruct the binding kinetics and thermodynamics, we used the inverse distances of the native contacts between antibody and antigen as input features for both the tICA and the Markov-state model. As a lag time, we chose both for the tICA as well as for the Markov-state model a lag time of 50 ns and defined 200 k-means clusters.

For quantitative analyses of the binding processes, the electrostatic and Van der Waals interactions were calculated with the lie, implemented in cpptraj. The images presented in this article were created using the PyMOL molecular graphics system (Schrodinger, 2015).

## Data Availability Statement

The original contributions presented in the study are included in the article/Supplementary Material, further inquiries can be directed to the corresponding author/s.

## Author Contributions

MF-Q performed research and wrote the manuscript. CS and PQ performed research and analyzed data. KL supervised the research. All authors contributed to writing the manuscript.

## Conflict of Interest

The authors declare that the research was conducted in the absence of any commercial or financial relationships that could be construed as a potential conflict of interest.
