# Supplementary material for: Shark Antibody Variable Domains Rigidify Upon Affinity Maturation—Understanding the Potential of Shark Immunoglobulins as Therapeutics
Source: Front Mol Biosci. 2021 Apr 20;8:639166. doi: 10.3389/fmolb.2021.639166 (PMC8093575; doi:10.3389/fmolb.2021.639166)
Supplement: Supplementary file 1 [file Table_1.docx]

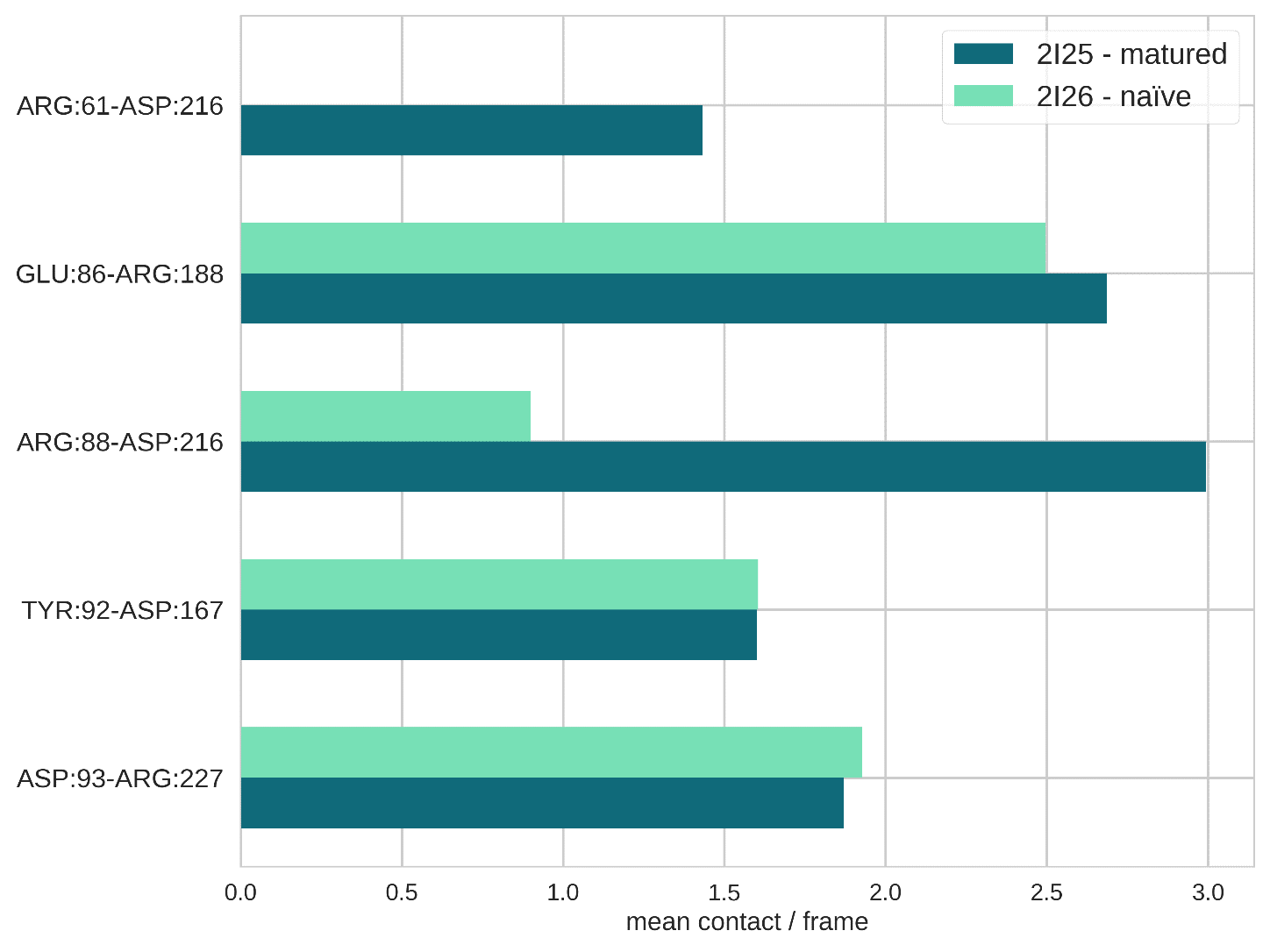


**SI Figure S1: Hydrogen bonds per frame for the naive (turquoise) and the matured (teal) V_NAR_.**


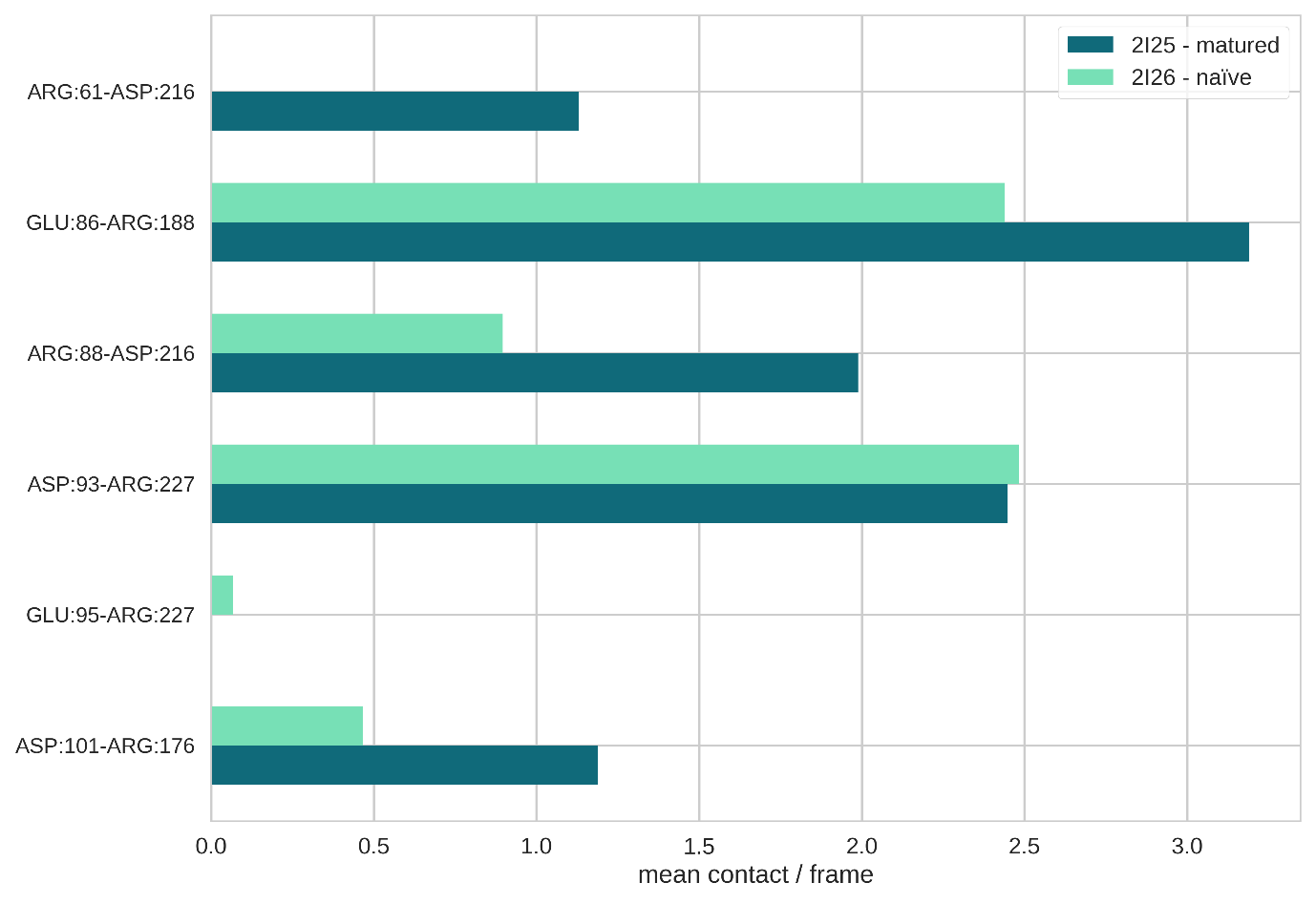


**SI Figure S2: Salt bridges per frame for the naive (turquoise) and the matured (teal) V_NAR._**

**
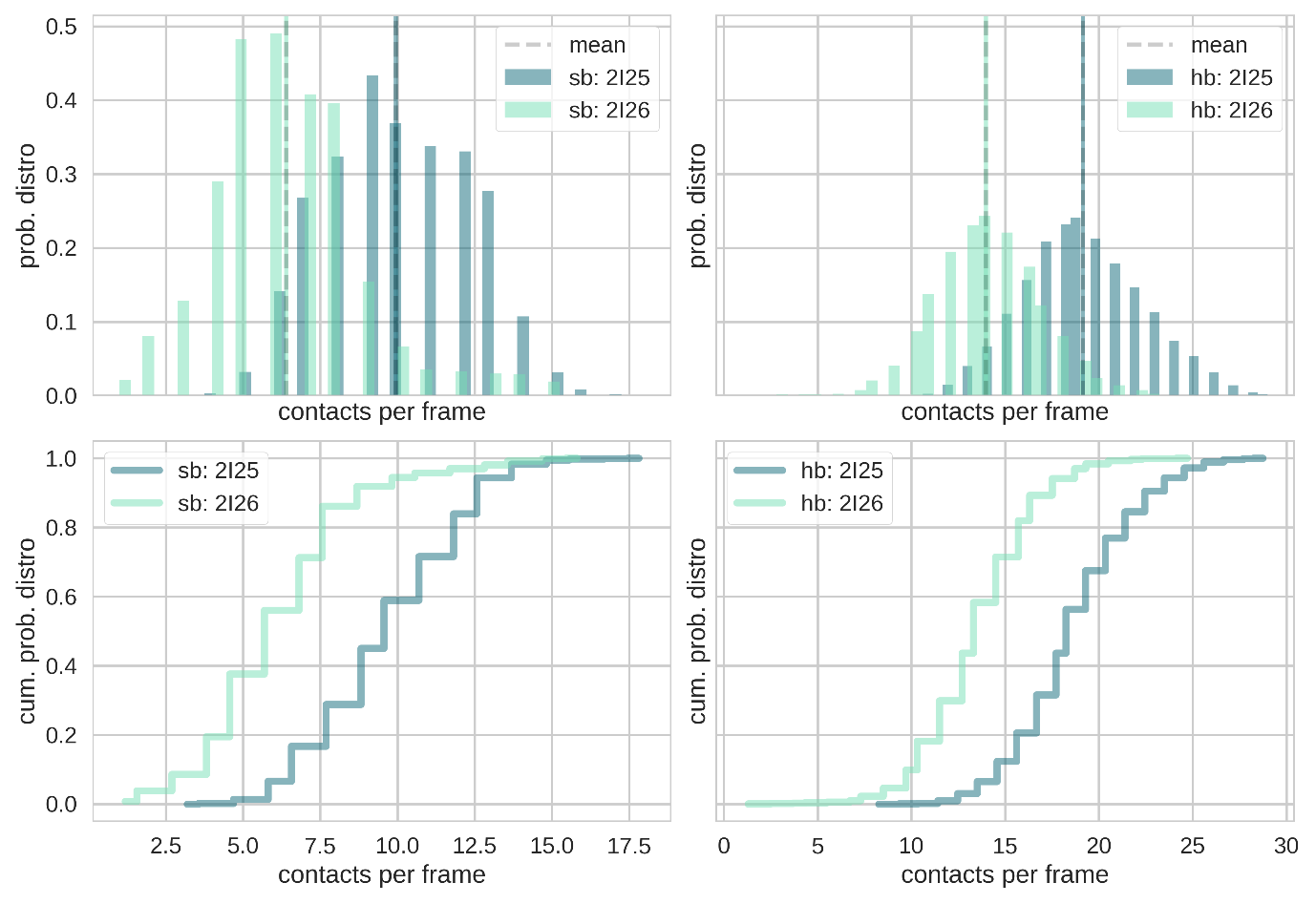
**

**SI Figure S3: Comparison of the distributions of salt bridges (left) and hydrogen bond (right) per frame. We show data of the naive and the matured V_NAR_s colored in turquoise and teal, respectively. While we show the probability distributions in the top panel, the cumulative distributions are depicted in the bottom panel. Herewith, we want to emphasize the difference between hydrogen bond and salt bridge distributions between the naive and the matured V_NAR_s.**
